# Endothelial nitric oxide synthase Asp298Glu (894G/T) gene polymorphism as a possible risk factor for the coronary slow flow phenomenon among Iranians

**DOI:** 10.1186/s12872-022-02736-0

**Published:** 2022-06-30

**Authors:** Yeganeh Karimi, Fatemeh Sehati, Ali Sarreshtedari, Mina Mirzad, Yasaman Khalili, Reza Kiani, Elham Taheri Bajgan, Maryam Hosseini Moghadam, Farzaneh Mehrvarz, Hooman Bakhshandeh, Maryam Parham, Mahshid Malakootian, Parham Sadeghipour

**Affiliations:** 1 Rajaie Cardiovascular Medical and Research Center, Iran University of Medical Sciences, Tehran, Iran; 2 Cardiovascular Intervention Research Center, Rajaie Cardiovascular Medical and Research Center, Iran University of Medical Sciences, Vali-Asr Ave, Niyayesh Blvd, Tehran, 1996911101 Iran; 3 Cardiogenetic Research Center, Rajaie Cardiovascular Medical and Research Center, Iran University of Medical Sciences, Vali-Asr Ave, Niyayesh Blvd, Tehran, 1996911101 Iran; 4 Molecular Genetics Department, Faculty of Biological Sciences, Tarbiat Modares University, Tehran, Iran

**Keywords:** Coronary artery disease, Endothelial dysfunction, Coronary slow flow phenomenon, Endothelial nitric oxide synthase, Interleukin-1β, rs1799983, rs1143634

## Abstract

**Background:**

Mounting evidence indicates an association between endothelial dysfunction and the coronary slow flow phenomenon (CSFP). In the present study, we aimed to evaluate the possible role of endothelial nitric oxide synthase (*eNOS*) 894G/T and interleukin-1β (*IL-1β*) 315C/T polymorphisms as possible risk factors for CSFP.

**Methods:**

This prospective study enrolled patients with CSFP and individuals with normal coronary arteries. Genotypes were assessed using regular polymerase chain reaction and direct Sanger-sequencing techniques.

**Results:**

The study population consisted of 267 individuals: 180 patients with CSFP (49 women [27.2%]) at a median age of 55 (48–62) years and 87 controls with normal coronary arteries (56 women [64.4%]) at a median age of 47 (41–58) years. The allelic distribution of *eNOS* 894G/T was significantly associated with CSFP (odds ratio [OR], 1.58; 95% confidence interval (CI), 1.04–2.42; *P* = 0.03). This polymorphism increased the risk of CSFP under the dominant model (OR 1.73; 95% CI I.02–2.95; *P* = 0.04). However, the allelic frequencies (1.05; 95% CI 0.68–1.59; *P* = 0.83) and genotypic frequencies (0.88; 95% CI 0.52–1.49; *P* = 0.63) of the IL-1β 315C/T polymorphism were not associated with the incidence of CSFP in the Iranian population.

**Conclusions:**

The CSFP and control groups were statistically different regarding the *eNOS* 894G/T polymorphism. Our findings also demonstrated that the *IL-1β* 315C/T polymorphism was not a risk factor for CSFP.

**Supplementary Information:**

The online version contains supplementary material available at 10.1186/s12872-022-02736-0.

## Introduction

The coronary slow flow phenomenon (CSFP), an uncommon disease, is an angiographic finding characterized by the delayed opacification of the distal branch of the coronary arteries in the absence of obstructive coronary artery disease [1, 2]. It is only found in 7% of patients with coronary artery disease undergoing diagnostic angiography [3]. CSFP seems to be multifactorial, and its precise etiopathological mechanisms have yet to be elucidated [4]. Morphological abnormalities such as fibromuscular hyperplasia, medial hypertrophy, myointimal proliferation, and subclinical atherosclerosis, as well as anatomic factors, functional abnormalities, and inflammation, have been proposed as the pathogenic factors of the disease [1, 4, 5]. Mounting evidence indicates common single-nucleotide polymorphisms (SNPs) residing in different genes as genetic risk factors for CSFP [6–9].

Nitric oxide (NO) is a vasodilator synthesized from L-arginine by endothelial nitric oxide synthase (*eNOS*), which is encoded by a single *eNOS* (*NOS3)* gene located on chromosome 7q35-q36 [10]. One of the most studied SNPs of *eNOS* is 894G/T (rs1799983), which results in the decreased production of NO and is significantly associated with coronary artery disease in different populations [11–17]. In addition, numerous studies have indicated that the plasma level of NO is significantly lower in patients with CSFP than in healthy controls [9, 18–20]. The interleukin-1 (IL-1) family comprises a group of proinflammatory cytokines composed of α and β types. The family, the product of the *IL-1* gene, modulates the chronic inflammatory response by increasing leukocyte adhesion to damaged endothelia, although several mediators are involved in the atherosclerosis process and cardiovascular disease [21].

The literature contains conflicting reports on the relationship between CSFP and *eNOS* 894G/T (rs1799983, Asp298Glu) and *IL-1β* 315C/T (rs1143634, Phe105 =) from studies carried out on different populations across the world [7, 9, 22–25].

In the present study, we sought to investigate the association between CSFP and *eNOS* 894G/T (rs1799983, Asp298Glu) and *IL-1β* 315C/T (rs1143634, Phe105 =) in a sample of the Iranian population, divided into patients with CSFP and normal individuals.

## Methods

### Study population

The study population was selected from candidates for coronary angiography in Rajaie Cardiovascular Medical and Research Center in Tehran, Iran. Patients with valvular heart disease, congenital heart disease, arrhythmia, connective tissue disease, collagen vascular disease, and more than 25% obstruction in the vessel diameter were excluded. The control group was chosen from individuals in whom diagnostic coronary angiography showed no coronary artery disease. Peripheral blood samples were taken from all the participants to determine genotypes, lipid profiles, cardiac enzyme levels, creatinine levels, cell blood counts, and erythrocyte sedimentation rates. The blood samples for genetic analysis were preserved at – 70 °C.

The study protocol was approved by the Ethics Committee of Rajaie Cardiovascular Medical and Research Center (IR.RHC.REC.1399.075), and the study was conducted in accordance with the Helsinki Declaration.

### Definition of CSFP

CSFP was diagnosed via the thrombolysis in myocardial infarction frame count (TFC) method.^1^ Participants with a corrected TFC greater than 2 standard deviations from the published normal range for the particular vessel were considered to have CSFP (the left anterior descending coronary artery (LADA) > 36.2 ± 2.6, the left circumflex artery (LCx) > 22.2 ± 4.1, and the right coronary artery (RCA) > 20.4 ± 3.0).

The standard method was drawn upon for left heart catheterization and coronary angiography. CSFP was defined based on the TFC method introduced by Gibson [26]. The number of cine frames required for the contrast to reach the standard landmark in the distal coronary artery is termed “TFC.” The first frame in TFC is obtained when the contrast material enters the coronary artery completely, with the entrance having 3 characteristics: (1) The contrast material should fill the full thickness of the vessel. (2) The contrast material should be in contact with both margins of the vessel. (3) The contrast agent should move forward. The last frame is obtained when the contrast material enters the distal landmark branch. The distal landmark branches are defined for each vessel separately: the last 2 branches for the left anterior descending, the last obtuse marginal branch for the left circumflex artery, and the first branch of the posterior left ventricular branch for the right coronary artery. The images were obtained at a rate of 15 frames per second, and the results were multiplied by 2. The frame counts of the left anterior descending were divided by 1.7 for correction because of its length. Patients who had a frame count above 27 for all vessels were considered to have CSFP.

### Genotyping of the *eNOS *and *IL-1β* gene polymorphisms

#### DNA extraction

Genomic DNA was extracted from the peripheral blood samples, collected in EDTA tubes, using the salting-out method and the Exgene Blood SV Mini Kit (GeneAll, Seoul, South Korea). The NanoDrop Spectrophotometer (Thermo Fisher Scientific, US) was employed to determine the quantity of the extracted DNA.

#### Polymerase chain reaction (PCR) and direct Sanger sequencing

Appropriate PCR oligonucleotides were designed to amplify the desired part of the *IL-1β* and *eNOS3* genes by utilizing the Gene Runner (Gene Runner 6.5.50) and PerlPrimer (PerlPrimer 1.1.21) software tools. Further, 5′-AAGGCAGGAGACAGTGGATG-3′ (forward), 5′-CAATTTCCAGCAGCATGTTG-3′ (reverse), 5′-CGTATATGCTCAGGTGTCCTC-3′ (forward), and 5′-CATGGAGAATTAGCAAGCTG-3′ (reverse) primers were used to amplify the part of *eNOS* (385 base pairs in length) and *IL-1β* (230 base pairs in length) that covered the desired variations with the following thermal program: 94 °C for 35 s, 63 °C (*eNOS*) or 55 °C (the *IL-1β* variant) for 30 s, and 72 °C for 45 s, with a final extension at 72 °C for 10 min. Amplicons were electrophoresed on a 1.5% agarose gel, stained with ethidium bromide, and visualized under ultraviolet light.

All the PCR products were subjected to direct Sanger sequencing with the ABI 3500 DNA Sequencer (Applied Biosystems, CA, US). The reverse primer of *eNOS3* and the forward primer of *IL-1β* were used for direct sequencing.

### Statistical analysis

HGMD [27], NCBI [28], UCSC [29], and VarSome [30] databases were utilized to evaluate the selected SNPs and the pathogenesis of the selected mutations.

The BioEdit software (BioEdit 7.2.1) was run to analyze the sequencing outcomes. The results were analyzed using the IBM SPSS statistics 26, the GraphPad Prism 9 software, and the SNPSTAT analyzer [31].

The 1-sample Kolmogorov–Smirnov test was first applied to test the normality of the data. Qualitative data were presented as numbers and percentages. The association between categorical variables was assessed using the χ^2^ test; and if 20% of the cells had the expected count of lower than 5, the Fisher exact test was employed. Quantitative data were described as the medians (Q_1_–Q_3_) for nonparametric data. The independent samples *t* test was applied to compare the mean values, and the Mann–Whitney test was used to compare the median values between 2 groups. Additionally, ANOVA and Kruskal–Wallis tests were drawn upon to compare the mean and median values between more than 2 groups. Finally, the multivariable regression analysis was applied using STATA 13.

## Results

### Clinical characteristics of the study population

From 2016 through 2017, a total of 180 patients with CSFP (49 women [27.2%]) at a median age of 55 (48–62) years were enrolled in the CSFP group. From 2016 through 2018, a total of 87 individuals with normal coronary arteries (56 women [64.4%]) at a median age of 47 (41–58) years were enrolled in the control group. The baseline and clinical characteristics of both groups are summarized in Table 1. Table 2 shows the clinical and laboratory characteristics of the CSFP group. Both groups were similar regarding baseline characteristics and laboratory data except for age and sex. The patient and control groups were similar in terms of dyslipidemia, diabetes mellitus, hypertension, smoking, and a family history for coronary artery disease. Additionally, the levels of plasma creatinine, triglyceride, and hemoglobin were higher in the patients with CSFP than in the control group. The median value (Q1–Q3) TFC for the left anterior descending coronary artery, the left circumflex artery, and the right coronary artery was 36 (29–43), 40 (31–50), and 31.5 (24–40), respectively, in the CSF group.

**Table 1:** Comparison of baseline characteristics and lab data between the CSFP and control groups^a,b^

Characteristics	Total (N = 267)	CSFP Group (n = 180)	Control Group (n = 87)	*P* value
Demographic				
Age (years)	52 (46–61)	55 (48–62)	47 (41–58)	< 0.001
Female/Male n (%)	105 (39.3)/ 162 (60.7)	49 (27.3)/ 131 (72.8)	56 (64.4)/ 31 (35.6)	< 0.001
Dyslipidemia (%)	87 (32.6)	63 (35)	24 (27.6)	0.41
Diabetes mellitus (%)	78 (29.2)	52 (28.9)	26 (29.9)	0.56
Hypertension (%)	118 (44.2)	76 (42.2)	42 (48.3)	0.12
Smoking status (%)	56 (21.0)	43 (23.9)	13 (14.9)	0.11
Family history for CAD^c^ (%)	74 (27.7)	55 (30.6)	19 (21.8)	0.26
Laboratory Tests^d^				
Fasting blood sugar (mg/dL)	106 (95–134.5)	109 (95–135)	102.5 (94.75–132.75)	0.37
Plasma creatinine (mg/dL)	1 (0.9–1.2)	1 (0.9–1.2)	0.9 (0.8–1)	< 0.001
Triglyceride (mg/dL)	128 (88–181)	140 (95.5–192)	111 (78–156)	< 0.01
Total cholesterol (mg/dL)	138.5 (116.25–163.75)	141 (116.5–168)	133 (115–155)	0.08
High-density lipoprotein-cholesterol (mg/dL)	38 (34–42)	38 (34.6–42)	37 (32–42)	0.33
Low-density lipoprotein-cholesterol (mg/dL)	76 (57–95)	77 (57–98)	72 (57–91)	0.26
Hemoglobin (g/dL)	14.1 (12.8–15.2)	14.2 (13.2–15.3)	13.2 (12.12–14.77)	< 0.001
White blood cell (cells/mm^3^)	6800 (5675–8125)	6800 (5700–7900)	6900 (5600–8400)	0.71
Platelet (× 10^**3**^/mm^3^)	219 (182–257.5)	216.5 (180–251.25)	225 (188.25–269.75)	0.15
Erythrocyte sedimentation rate (mm/h)	10 (5–17)	10 (5–15)	12 (6–18.75)	0.04

CSFP: coronary slow flow phenomenon; CAD: coronary artery disease

^a^Continuous variables are presented as the median (Q_1_–Q_3_)

^b^Categorical variables are presented as numbers (%)

^c^Family history of coronary artery diseases (CAD): the presence of CAD in a first-degree male or female relative before age 55 or 65 years, respectively

^d^Normal ranges of the measured lab tests were defined as follows: < 100 mg/dL for fasting blood sugar, 0.6–1.4 mg/dL for plasma creatinine, < 150 mg/dL for triglyceride, < 200 mg/dL for total cholesterol, > 60 mg/dL for high-density lipoprotein, < 110 mg/dL for low-density lipoprotein, 13.5–17.5 g/dL for men and 12–15.6 g/dL for women for the hemoglobin level, 4500–11,000 cells/mm^3^ for the white blood cell count, 150–450 10^3^/fL for the platelet count, < 22 mm/h for men and 29 mm/h for women for the erythrocyte sedimentation rate

**Table 2:** Clinical, laboratory, and angiographic characteristics of the CSFP group^a,b^

Characteristics	Patients
*Clinical*	
Chest pain	
Typical	107 (59.4)
Atypical	59 (32.8)
Dyspnea	58 (32.2**)**
Palpitation	8 (4.4)
Past medical history	
Prior PCI	14 (7.8)
Prior MI	10 (5.6)
HF	1 (0.6)
BMI^c^	28.3 (25.7–30.97)
Baseline hemodynamics	
Systolic blood pressure (mm Hg)	125 (119–135)
Diastolic blood pressure (mm Hg)	80 (70–80)
Laboratory and Echocardiography Characteristics	
hs-CRP	1.7 (0.7–4)
Positive cardiac enzyme	24 (13.3)
LVEF	50 (50–55)
*Angiographic characteristics*	
TFC	
Left anterior descending artery	36 (29–43)
Left circumflex artery	40 (31–50)
Right coronary artery	31.5 (24–40)
Single-vessel slow flow coronary artery	15 (8.3)
Double-vessel slow flow coronary arteries	57 (31.7)
Triple-vessel slow flow coronary arteries	108 (60)

*CSFP* coronary slow flow phenomenon, *PCI* percutaneous coronary intervention, *MI* myocardial infarction, *HF* heart failure, *BMI* body mass index, *hs-CRP* high-sensitivity C-reactive protein, *LVEF* left ventricular ejection fraction, *TFC* thrombolysis in myocardial infarction frame count

^a^Continuous variables are presented as the median (Q_1_–Q_3_)

^b^Categorical variables are presented as numbers (%)

^c^Weight (in kilograms) divided by the square of the height (in meters)

### Allelic and Genotype Distributions of* eNOS *894G/T and *IL-1β* 315C/T

The results concerning the allelic and genotype distributions of the *eNOS3* and *IL-1β* polymorphisms are depicted in Table 3. The genotype frequencies of eNOS 894G/T polymorphism was in accordance with the Hardy–Weinberg equilibrium in the CSFP group (χ2 = 1.484, *P* = 0.48) and in control group (χ2 = 0.1867, *P* = 0.91). Likewise, the genotype frequencies of IL-1β 315C/T polymorphism was in line with those predicted by the Hardy–Weinberg equilibrium in the CSFP group (χ2 = 0.3557, *P* = 0.84) and in control group (χ2 = 6.446, *P* = 0.04).

**Table 3:** Distributions of the eNOS3 864G/T and IL-1β 315C/T alleles and genotypes in the CSFP and control groups

	Patients With CSFP; N (%)	Controls; N (%)	OR (95% CI)^b^	*P* value^a^
*eNOS 894G/T*				
Allele Frequency				
G	287 (80)	124 (71)	1.00	0.03
T	73 (20)	50 (29)	1.58 (1.04–2.42)	
Total	360 (100)	174 (100)		
Genotypes (codominant)				
G/G	117 (65)	45 (51.7)	1.00	0.11
G/T	53 (29.4)	34 (39.1)	0.60 (0.35–1.04)	
T/T	10 (5.6)	8 (9.2)	0.48 (0.18–1.30)	
Total	180 (100)	87 (100)		
Genotypes (dominant)				
G/G	117 (65)	45 (51.7)	1.00	0.04
G/T-T/T	63 (35)	42 (48.3)	1.73 (1.02–2.95)	
Genotypes (recessive)				
G/G-G/T	170 (94.4)	79 (90.8)	1.00	0.27
T/T	10 (5.6)	8 (9.2)	1.72 (0.66–4.68)	
HWE^c^	*X*^2^ = 1.484, *P* = 0.48	*X*^2^ = 0.1867, *P* = 0.91		
*IL-1β 315C/T*				
Allele Frequency				
C	269 (76)	130 (75)	1.00	0.83
T	87 (24)	44 (25)	1.047 (0.68–1.59)	
Total	356 (100)	174 (100)		
Genotype(codominant)				
C/C	103 (57.9)	53 (60.9)	1.00	0.25
C/T	63 (35.4)	24 (27.6)	1.73 (0.84–3.56)	
T/T	12 (6.7)	10 (11.5)	0.81 (0.26–2.52)	
Total	178 (100)	87 (100)		
Genotype(dominant)				
C/C	103 (57.9)	53 (60.9)	1.00	0.63
C/T-T/T	75 (42.1)	34 (39.1)	0.881 (0.52–1.49)	
Genotype (recessive)				
C/C–C/T	166 (93.3)	77 (88.5)	1.00	0.19
T/T	12 (6.7)	10 (11.5)	1.797 (0.73–4.10)	
HWE^c^	*X*^2^ = 0.3557, *P* = 0.84	*X*^2^ = 6.446, *P* = 0.04		

*CSFP* coronary slow flow phenomenon

^a^Significant *P* values if ≤ 0.05

^b^OR: odds ratio, 95% CI 95%: confidence interval

^c^Hardy–Weinberg equilibrium

In the univariate analysis of *eNOS* 894G/T, the frequencies of the T allele in the patient and control groups were 20% and 29%, respectively, and a significant difference was found in the allelic distribution of *eNOS* (odds ratio [OR], 1.58; 95% confidence interval [CI], 1.04–2.42; *P* = 0.03). Consequently, the higher presence of the T allele of *eNOS* in the control group hinted at a protective effect exerted by this allele on the study population. In the CSFP and control groups, respectively, the frequency of the G/G genotype was 65% versus 51.7%, the frequency of the G/T genotype was 29.4% versus 39.1%, and the frequency of the T/T genotype was 5.6% versus 9.2%. The analysis of the genotype distribution in the 2 groups demonstrated a significant association between the presence of the T allele of *eNOS* and CSFP (OR 1.73; 95% CI 1.02–2.95; *P* = 0.04) in a dominant model. Nonetheless, no significant differences were found between the recessive model and the codominant model.

In the univariate analysis of *IL-1β* 315C/T, the frequency of the T allele was estimated to be 24% and 25% in the patients and controls, respectively, and no significant difference was found in the allelic distribution of *IL-1β* (*P* = 0.83). The frequencies of the C/C, C/T, and T/T genotypes were 57.9% versus 60.9%, 35.4% versus 27.6%, and 6.7% versus 11.5% in the patients with CSFP and the participants with normal coronary arteries, respectively (*P* = 0.25). Additionally, no significant differences in genotype distribution were found in the dominant (*P* = 0.63), recessive (*P* = 0.19), and codominant (*P* = 0.25) models between the patients and the healthy controls concerning *IL-1β*.

Furthermore, the association between the CSFP phenotype and the combined genotypes of the *eNOS* and *IL-1β* polymorphisms was assessed, and the results were nonsignificant (*P* = 0.12) (data not shown).

In the multivariable regression analysis, age (OR 1.08; 95% CI; 1.03 to 1.12; *P* < 0.01) and the male sex (OR 0.22; 95% CI 0.08 to 0.62; *P* < 0.01) were the only independent predictors of CSFP in the study population. In addition, no significant associations were found between the presence of the mutant allele and the wild type for the *eNOS* and *IL-1β* polymorphisms with the application of the multivariable analysis (OR 0.46; 95% CI 0.11 to 1.98; *P* = 0.29 for *eNOS*, and OR 0.47; 95% CI 0.13 to 1.7; *P* = 0.24 for *IL-1β*) (Table 4).

**Table 4:** Multivariable logistic regression analyses of the possible predictors of CSFP in the study population

Variables	OR (95% CI)	*P* value
Presence of allele ‘T’ of the *eNOS* 864G/T polymorphism	0.46 (0.11–1.98)	0.29
Presence of allele ‘T’ of the *IL-1β* 315C/T polymorphism	0.47 (0.13–1.7)	0.24
Interaction of *eNOS* 864G/T and *IL-1β* 315C/T	1	
Age	1.08 (1.03–1.12)	< 0.01
Gender	0.22 (0.08–0.62)	< 0.01
Smoking	1.17 (0.48–2.83)	0.73
FH of CAD	2.15 (0.92–5.03)	0.07
DLP	1.16 (0.51–2.63)	0.72
DM	0.49 (0.19–1.25)	0.13
HTN	0.74 (0.33–1.64)	0.45
FBS	1.0 (0.99–1.01)	0.15
Cr	2.51 (0.4–15.77)	0.32
LDL-cholesterol	1.01 (1–1.03)	0.05
Hb	1.10 (0.81–1.50)	0.52
WBC	1 (1–1)	0.96
Platelet (× 10^**3**^)	1.0 (0.99–1.01)	0.79
ESR	1.01 (0.96–1.05)	0.77

*CSFP* coronary slow flow phenomenon, *FH of CAD* family history of coronary artery disease, *DLP* dyslipidemia, *DM* diabetes mellitus, *HTN* hypertension, *FBS* fasting blood sugar, *Cr* creatinine, *LDL* low-density lipoprotein, *Hb* hemoglobin, *WBC* white blood cell, *ESR* erythrocyte sedimentation rate

### Relationships Between the *eNOS3* 864G/T and *IL-1β* 315C/T Genotypes and TFC and Electrocardiographic Findings in the CSFP Group

According to the univariate analysis, the median values of TFC for the left anterior descending (*P* = 0.80), the left circumflex (*P* = 0.16), and the right coronary artery (*P* = 0.80) were not significantly different between the individuals with different genotypes of *eNOS* 894G/T. Moreover, no significant differences were found in terms of the median values of TFC for the left anterior descending (*P* = 0.53) and the left circumflex (*P* = 0.11) between the genotypes of *IL-1β* 315C/T. However, the median value of TFC for the right coronary artery was different between the *IL-1β* genotypes (*P* < 0.01; Table 5).

**Table 5:** Relationships between the *eNOS3* 864G/T and *IL-1β* 315C/T genotypes and TFC findings of the CSFP group^a^

TFC, median (Q_1_–Q_3_)	*eNOS* 894G/T				*IL-1β* 315C/T			
	G/G	G/T	T/T	*P* value^b^	C/C	C/T	T/T	*P* value^b^
LAD	35 (29–45)	37.5 (29–43.75)	37.5 (29–51.75)	0.80	38 (30–43.5)	35 (29–41)	37.5 (29–51.75)	0.53
LCx	40 (32–50)	37 (30–48)	46 (36–61.5)	0.16	40 (32–50)	38 (30–44)	46 (36–61.5)	0.11
RCA	32 (24–40)	30 (23.5–40)	25.5 (16.5–28)	0.80	32 (25.5–41)	30 (22–40)	25.5 (16.5–28)	< 0.01

*TFC* thrombolysis in myocardial infarction frame count, *LAD* left anterior descending artery, *LCx* left circumflex artery, *RCA* right coronary artery

^a^Categorical variables are presented as numbers (%)

^b^Significant *P* values if ≤ 0.05

Sex (coefficient, − 3.48; 95% CI − 7.06 to 0.11; *P* = 0.05) was the only predictor of TFC for the left anterior descending, and no statistically significant associations were found between TFC for the left anterior descending and the presence of the T allele of the *eNOS* 864 T/G polymorphism, the presence of the mutant allele of the *IL-1β* 315C/T polymorphism, body mass index, systolic blood pressure, and left ventricular ejection fraction in the multivariable regression analysis (*P* > 0.05) (Additional file 1: Table S1).

Further, all 180 patients with CSFP underwent electrocardiography. Among them, ST-T changes were positive in 43 patients (23.9%), of whom 17 (39.5%) had the T allele in the *eNOS3* locus. No significant differences were noted in electrocardiographic findings between the *eNOS3* genotypes.

Similar to the *eNOS* results, no significant associations were found in electrocardiographic findings between the different genotypes of the studied *IL-1β* SNP. The distribution of the *IL-1β* genotypes was similar among those with a positive ST-T change finding (*P* = 0.26).

## Discussion

In the present study, we examined the association between CSFP and *eNOS3* (894G/T) and *IL-1β* (315C/T) polymorphisms in a sample of the Iranian population. Our results indicated that the distribution of the Asp298Glu variant of the *eNOS* gene was significantly different between patients with CSFP and controls with normal coronary arteries. Further, the mutant allele T of *eNOS* 894G/T polymorphism was lower in the CSFP suggesting that this polymorphism is protective. While there was no significant association between the *IL-1β* gene (315C/T) variant and CSFP in our studied population. We also assessed associations in diagnostic tests, clinical information, and lab data between the *eNOS3* (894G/T) and *IL-1β* (315C/T) variants and found no significant associations.

CSFP was first defined by Tambe et al. in 1972 as a delay in the progression of the contrast dye injected into the coronary arteries during coronary angiography without any obstructive disease [32]. The phenomenon is diagnosed mainly with an increased TFC. Although the etiology and pathogenesis of CSFP are not well-known, impaired balances between vasoconstrictor and vasodilator factors and increased inflammatory markers have been suggested [4, 9, 33]. Urotensin-II, as a potent vasoconstrictor, has been reported as a possible risk factor for CSFP (OR 1.01; 95% CI 1.00–1014; *P* = 0.01) [34]. Furthermore, aortic pulse pressure and the pulsatility index in patients with CSFP tend to rise remarkably due to endothelial dysfunction. The role of inflammation in the pathophysiology of CSFP was expounded by Aksn G et al., who found that the serum levels of neutrophil gelatinase-associated lipocalin, as an inflammatory biomarker, were significantly higher in patients with CSF than in those with a normal coronary flow [35]. In addition, the hematocrit level, as well as erythrocyte, eosinophil, and basophil counts, was increased in patients with CSF compared with the group with a normal coronary flow, which may support the previous hypothesis [36]. Substantial evidence suggests that the *eNOS* Glu298Asp polymorphism is responsible for endothelial dysfunction [37–39].

NO plays a significant role as a vasorelaxation factor and has a protective effect on atherogenesis [40]. It has been shown that several polymorphisms of *eNOS (NOS3)* affect the serum level of NO [41]. Notably, the *eNOS* Asp298Glu polymorphism may be associated with CSFP in that it decreases the serum levels of NO. Moreover, the *IL-1β* gene, which releases *IL-1β* as a proinflammatory agent, is associated with cardiovascular diseases, including coronary artery disease, stent restenosis after percutaneous coronary interventions, carotid artery disease, lone atrial fibrillation, and CSFP [42–45]. In addition, the 315C/T nucleotide transition of the *IL-1β* gene probably modulates IL-1β protein synthesis and is associated with such cardiovascular diseases as CSFP, coronary artery disease, and myocardial infarction [25, 46–49].

Previous studies have also indicated the role of genetic predisposing factors in the occurrence of CSFP [22–24, 50].

There are dissimilarities in the frequencies of *eNOS3* 894G/T alleles in different races. Such differences have given rise to controversy as regards the application of the G allele as a mutant. The VarSome database recognizes the T allele as the reference allele, and the Iranome database also cites the same allele for the Iranian population. [51, 52]. However, the T allele has been reported as a possible risk factor for stroke and periventricular white matter hyperintensities [53, 54]. Marwa Ben et al. concluded that *eNOS3* 894G/T was significantly associated with coronary artery disease in additive and dominant models (but not in recessive models), concordant with our findings [55]. In Pakistan, Nawaz et al. reported that the frequency of the T allele was higher than that of the G allele and introduced the TT genotype as a strong risk factor for coronary artery disease [56]

Controversy, however, abounds regarding the association between CSFP and *eNOS3* 894G/T SNPs in different populations. In samples of the Turkish population, Caglayan et al. and subsequently Sezgin et al. reported no associations between 894G/T SNP and CSFP [9, 57]. Caglayan and colleagues assessed 85 individuals, consisting of 66 patients with CSFP and 19 subjects with normal coronary arteries, while they excluded patients with diabetes mellitus; hypertension; coronary artery disease history; coronary ectasia; atrial fibrillation; complete bundle branch block; serious conduction defects; mitral valve prolapse; hypertrophic, restrictive, and dilated cardiomyopathies; left ventricular hypertrophy; ejection fractions less than 50%; and pulmonary, renal, hepatic, and hematological disorders. In this study, the frequency of the variant allele was 0.41 and 0.38 in the control and patient groups, respectively. No statistically significant differences were found in allelic and genotype distributions between the CSFP and control groups. Sezgin and colleagues recruited 30 patients with CSFP and no other cardiac disease and 61 control subjects and reported no association between *eNOS* intron 4 VNTR and 894G/T polymorphisms. Nevertheless, the plasma levels of NO were significantly lower in the CSFP group than in the control group (*P* < 0.05). In contrast, Gupta et al. reported a strong association between this nucleotide transition and CSFP in the North Indian population and suggested the T allele as an independent risk factor for CSFP [24]. This study assessed 27 patients with CSFP and 200 individuals as the control group. The exclusion criteria were the same as those in the study by Caglayan and colleagues. The results showed a significant association between the presence of the T allele and CSFP (*P* = 0.014; w^2^ = 6.1). Our findings are different from those reported by the investigations in Turkey, but they chime in with those reported by Gupta and colleagues.

Mutluer et al. revealed an association between the rs1143634 of the *IL-1β* gene and CSFP in the Turkish population [48]. A study on the Han Chinese population reported an association between the *IL-10* polymorphism and CSFP [45]. In contrast to the investigation in the Turkish population, our results showed no association between the 315C/T (rs1143634) of the *IL-1β* gene polymorphism and CSFP. It is worthy of note that had we recruited a larger population, our analysis might have yielded different results. To the best of our knowledge, this is the first report on the association between *eNOS* 894G/T and CSFP in the Iranian population. A previous investigation in Iran examined the predictive power of 2 common polymorphisms of the *eNOS* gene in relation to CSFP after primary percutaneous coronary interventions and reported no associations between CSFP and the 894G/T and − 786T/C polymorphisms of the *eNOS* gene [58]. Heidari et al. found an association between the − 813C/T (rs2070744) and 894G/T (rs1799983) polymorphisms of the *eNOS* gene and multiple sclerosis in Iranian patients [59]. In another study, no association was found between the 894G/T *eNOS* polymorphism and coronary artery disease in the northern Iranian population [60]. Accumulating evidence indicates that the Asp298Glu SNP of the *eNOS* gene is associated with coronary artery disease, ST-segment-elevation myocardial infarction, hypertension, coronary vasospasm, impaired coronary collateral development, impaired coronary blood flow, and obesity [14, 61–67].

### Limitations

The observational nature of our investigation and its limited sample size precluded us from drawing a firm conclusion. Indeed, our results should be tested in a larger population to confirm the association between the studied *eNOS* gene polymorphisms and the *IL-1β* nucleotide transition. Additionally, the associations between the Asp298Glu transition of the *eNOS* gene and the plasma nitric oxide level and nitric oxide synthase activity were not assessed in this study due to technical and financial limitations. Finally, our results might have been influenced by dissimilarities between the patient and control groups.

## Conclusions

The present preliminary study is the first to suggest an association between the 894G/T *eNOS* gene polymorphism and CSFP in the Iranian population. However, our results demonstrated no association between CSFP and the 315C/T *IL-1β* gene variant. Further, the allelic distribution and the presence of the variant allele of the 894G/T *eNOS* gene polymorphism were statistically associated with CSFP.

## Supplementary Information

**Additional file 1:** Multivariable regression analysis between the TIMI frame count for the LAD and clinical and genetic parameters in the patients.

## Data Availability

All the oligos and the information regarding the study are provided in the paper. The accession numbers of the nucleotide transitions are 894G/T (rs1799983) for the *eNOS* change and 315C/T (rs1143634) for the *IL-1β* change (https://www.ncbi.nlm.nih.gov/search/all/?term=dbsnp). HGMD (http://www.hgmd.cf.ac.uk/ac/index.php), NCBI (www.ncbi.nlm.nih.gov/clinvar), UCSC (https://genome.ucsc.edu), and VarSome (https://varsome.com/) databases were utilized to investigate the selected SNPs and the pathogenesis of the selected nucleotide variations. The accession numbers and all the repositories used for the study are both mentioned in the article and declarations part as well.
