# Supplementary material for: Endothelial nitric oxide synthase Asp298Glu (894G/T) gene polymorphism as a possible risk factor for the coronary slow flow phenomenon among Iranians
Source: BMC Cardiovasc Disord. 2022 Jun 30;22:300. doi: 10.1186/s12872-022-02736-0 (PMC9248196; doi:10.1186/s12872-022-02736-0)
Supplement: Supplementary file 1 — Additional file 1: Multivariable regression analysis between the TIMI frame count for the LAD and clinical and genetic parameters in the patients. [file 12872_2022_2736_MOESM1_ESM.docx]

**Supplementary table**

**Supplementary Table 1**: Multivariable regression analysis between the TIMI frame count for the LAD and clinical and genetic parameters in the patients ^a b^

| **Variables** | ***P* value** ^c^ | **Coefficient** | **95%CI** |
| --- | --- | --- | --- |
| Sex | 0.05 | -3.48 | -7.06,0.11 |
| Body mass index | 0.06 | 0.35 | -0.01, 0.72 |
| Systolic blood pressure | 0.19 | -0.07 | -0.17, 0.03 |
| Left ventricular ejection fraction | 0.47 | -0.09 | -0.34, 0.16 |
| Presence of allele ‘T’ of the eNOS 864G/T polymorphism | 0.69 | 1.31 | -5.3, 7.92 |
| Presence of allele ‘T’ of the IL1b 315C/T polymorphism | 0.34 | 3.10 | -3.40, 9.61 |
| 95%CI-95% confidence interval; BMI-body mass index; SBP-systolic blood pressure; LVEF-left ventricular ejection fraction.  ^a^ Continuous variables are presented in median (Q1,Q3).  ^b^ Categorical variables are presented in number (%).  ^c^ Significant P values if ≤0.05. | | | |
